# Supplementary material for: Effect evaluation, prediction and response strategy analysis of China’s birth policy adjustment
Source: PLoS One. 2025 Sep 18;20(9):e0330308. doi: 10.1371/journal.pone.0330308 (PMC12445481; doi:10.1371/journal.pone.0330308)
Supplement: Attachment — Submitted filename: Comments.docx [file pone.0330308.s001.docx]

Thank you for the opportunity to review the paper. Its subject is both interesting and highly relevant, particularly given China’s current context as one of the countries with the lowest fertility rates globally. With government efforts underway to raise fertility rates through various population policies, this research addresses an important issue. However, some revisions are needed to prepare the paper for publication.

1) Written and Editorial Issues

- Figure Titles: None of the figures have titles, and the quality of the images is low.
- Explanation of Figures: The explanation above Figure 3 (age pyramid) is unclear and confusing.
- Figures 4, 5, and 6 lack accompanying explanations in the text.
- Figures 4 and 5 contain axis in Chinese, which needs translation for consistency.
- The last figure should be labeled as Figure 7 (currently labeled as Figure 6 in the text). It is also unclear which scenario each color represents within this figure.
- In Table 2, “fertility rate” is mentioned in both of the first two rows, but one of them is incorrect.
- in Table 1: In the last two rows of control Variables, the terms are unclear. The variable name appears as “support ratio,” but the definition provided aligns with the “dependency ratio.” In the support ratio, the denominator should include the dependents, with working-age population in the numerator.

2) Conceptual and Content Issues

- Content currently in section 3.1 would be better placed in the introduction, particularly to help readers unfamiliar with China’s demographic profile. Additionally, there is overlap in some of the material, which could be streamlined. This adjustment is essential, as readers without knowledge of China’s fertility trends do not encounter this information until page 4.
- IMPORTANT: The terms used are inconsistent or imprecise, for example:
  - “Birth population” should be replaced with “Birth number.”
  - The term “birth rate” is inaccurately applied; if it refers to CDR, this rate should be defined as the number of births per thousand people, not as a percentage, as it is currently presented in Figure 1 and the text.
- The methodology used for predictions is unclear, and each scenario’s assumptions are not explained. The only detail provided is that none of the forecasts suggest a future increase in births, but further clarification is needed.
- In the conclusion, the findings should be contextualized alongside relevant theoretical concepts (such as Becker’s theory).
- The language used to present suggestions should be revised for clarity and tone.
