# Supplementary material for: Effect evaluation, prediction and response strategy analysis of China’s birth policy adjustment
Source: PLoS One. 2025 Sep 18;20(9):e0330308. doi: 10.1371/journal.pone.0330308 (PMC12445481; doi:10.1371/journal.pone.0330308)
Supplement: Attachment — Submitted filename: Response to Reviewers.docx [file pone.0330308.s002.docx]

**Response to Reviewers**

Manuscript ID: [PONE-D-24-44118]

Title: Effect evaluation, prediction and response strategy analysis of China’s birth policy adjustment

Dear Editor and Reviewers,

We sincerely appreciate your meticulous review and constructive comments. Please find below our point-by-point responses to your suggestions, along with an important authorship update.

**I. Authorship Change Notification**

To ensure proper attribution of contributions, we propose the following changes to the authorship list:

1. New Corresponding Author:

Yalan Mo (Lecturer, Guilin Tourism University)

Contributions:

✓ Led the PRISMA-based literature review update (Section 2.3)

✓ Processed NSFC microdata for mediation analysis (β = 0.32 results)

✓ Formulated the progressive subsidy policy framework (Section 5.4)

2. New Third Author:

Yanxi Kuang (MSc candidate, Guilin University of Electronic Technology)

Contributions:

✓ Designed robustness tests (Placebo & Randomization tests in Section 4.2)

✓ Created all robustness test visualizations (Figures 7-8)

✓ Standardized demographic terminology (e.g., "TFR" definition)

All new authors have approved:

- The final manuscript version

- Authorship order

(Attached: Signed authorship change form + ORCIDs)

**II. Revisions Summary**

A. Typographical/Editorial Issues

✓ Added complete figure titles/axis labels (Figs.1-8)

✓ Standardized variables:

- "support ratio" → "dependency ratio" (Table 2)

- "birth population" → "Birth Number" (with units)

B. Conceptual Enhancements

1. Restructured Introduction:

- Integrated Section 3.1 content into background

- Added unresolved debates (e.g., urban-rural policy effect heterogeneity)

2. Revised Literature Review:

- New taxonomy: "Policy Effects → Economic Constraints → Institutional Regulation"

- Added "Theoretical Integration" subsection linking Becker model to DID design

3. Terminology Precision:

- Uniformly use "total fertility rate (TFR)" with definition

C. Methodological Improvements

✓ New Robustness Tests:

- Time placebo test (p > 0.1 in Fig.7)

- Random treatment assignment test (p > 0.1 in Fig.8)

✓ Mediation Analysis:

Quantified "childcare services → fertility intention" path (β = 0.32***)

D. Policy Recommendations

✓ Proposed OECD-inspired graduated subsidies :

5%/8%/12% of income for 1/2/3-child families

✓ Added ILO-aligned parental leave standards

Attachments

1. Revised Manuscript (Track Changes)

2. Clean Manuscript Version

3. Authorship Change Documentation

We believe these revisions have significantly strengthened the study's rigor and policy relevance. Should any further modifications be required, we stand ready to comply promptly.

Best regards,

Wei Wang

On behalf of all authors

June 3, 2025
